# Proteins of Unknown Function in the Protein Data Bank (PDB): An Inventory of True Uncharacterized Proteins and Computational Tools for Their Analysis

**DOI:** 10.3390/ijms131012761

**Published:** 2012-10-08

**Authors:** Nurul Nadzirin, Mohd Firdaus-Raih

**Affiliations:** School of Biosciences and Biotechnology, Faculty of Science and Technology, Universiti Kebangsaan Malaysia, 43600 UKM Bangi, Malaysia; E-Mail: nurul@mfrlab.org

**Keywords:** Protein Data Bank, proteins of uncharacterized function, proteins of unknown function, structural similarity, 3D motifs

## Abstract

Proteins of uncharacterized functions form a large part of many of the currently available biological databases and this situation exists even in the Protein Data Bank (PDB). Our analysis of recent PDB data revealed that only 42.53% of PDB entries (1084 coordinate files) that were categorized under “unknown function” are true examples of proteins of unknown function at this point in time. The remainder 1465 entries also annotated as such appear to be able to have their annotations re-assessed, based on the availability of direct functional characterization experiments for the protein itself, or for homologous sequences or structures thus enabling computational function inference.

## 1. Introduction

The Protein Data Bank (PDB) remains the largest repository of experimentally determined biological macromolecular structures [1] with records in excess of 80,000 entries that comprise of proteins, nucleic acids and complex assemblies. The majority of the PDB’s structures are proteins and approximately 14% of these were solved as part of structural genomics initiatives. A not unsubstantial proportion of these protein structures were designated as proteins of unknown function because of no detectable homology to proteins of known functions at both the sequence and structure level [2], or the functions have not been characterized from assays following the structural analyses. The lack of information on a protein’s characterized function restricts the further utility of these proteins.

**Figure 1:**
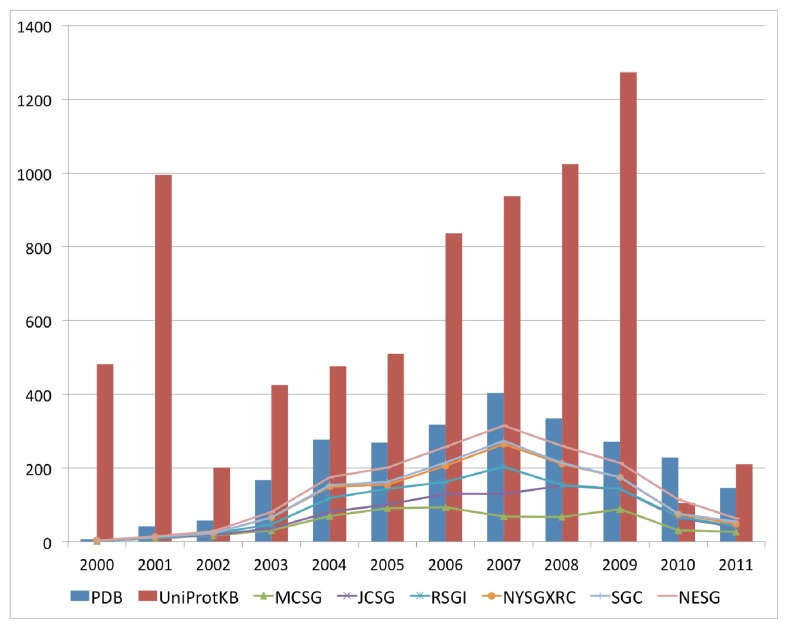
The bars show the trend of uncharacterized protein sequence (in UniProtKB) and structure (in the Protein Data Bank (PDB)) determination from 2000 to 2011. The stacked lines show uncharacterized protein structures in the PDB solved by the top six Structural Genomics Centers from 2001 to 2011, in comparison with structures solved by other laboratories or institutions. The top six centers are RIKEN Structural Genomics/Proteomics Initiative (RSGI), Midwest Center for Structural Genomics (MCSG), Joint Center for Structural Genomics (JCSG), Structural Genomics Consortium (SGC), New York Research Center for Structural Genomics (NYSGXRC), and Northeast Structural Genomics Consortium (NESG).

The past decade has seen an increase in the number of deposited structures for proteins of unknown functions that can perhaps be mainly attributed to the availability of numerous completed and draft genomes, which enable complete open reading frames to be selected as targets for structure solution as either a part of high throughput structural genomics approaches, or as follow up investigations to functional genomics analysis of gene expression data. This marked increase in the deposition of structures with uncharacterized functions began in the year 2000 and peaked in 2007 for the 2000–2011 period (Figure 1). This period coincided with an increase in the availability of sequences for uncharacterized proteins in available sequence databases, resulting from the completion of various genome-sequencing projects for a variety of bacterial, archaeal and eukaryotic genomes. The top six structural genomics centers contributed up to 69% of the total number of uncharacterized protein structures in the PDB (Figure 1). Although there is a downtrend with regard to the volume of deposits annually for uncharacterized protein structures, their numbers remain high.

Progress made in determining the biological function of proteins through experimental characterization has not always been correlated to the body of uncharacterized protein sequence and structure data. As a result, the discovery and assignment of biological function for a protein with previously unknown function is not actively tracked for the purposes of updating annotations associated to protein structure data in the PDB, and lags behind the rate which the assignments of uncharacterized proteins are being carried out. Therefore, as the volume of uncharacterized protein structure data grows larger, there arises a need for the systematic and periodical revision of PDB data to be updated with newly characterized functions as and when they become available.

As a result of this scenario, the PDB also contains proteins of unknown function that can either be said as misattributed or should be reviewed in the sense that either (i) their functions have been directly determined or (ii) by virtue of possessing strong similarities to proteins of known functions at the sequence or structure level, their functions can be inferred. The reason why such structures are accumulating is perhaps a compounding effect of the PDB deposition and maintenance process. Details of the PDB deposition process has been previously reported [3]; briefly, deposition is initiated when the coordinate data, as well as experimental and bioinformatics functional analyses are provided to the PDB team. After the validation and correction process, the structure is made public with a unique PDB ID on a specific deposition date. Corrections or re-annotations succeeding this date are e-mailed to the RCSB team, and the entry will then be modified during the weekly PDB update [4]. However, it is clear from our analysis that structures annotated as proteins of unknown function may actually possess sequence or structural homology with the current set of proteins with known functions, but this has gone unreported.

In order to propose a potential mechanism through which the PDB can be periodically revised for function updates, we investigated the current extent of this problem in the PDB. Using standard sequence and structure homology detection tools, we were able to provide an accounting of the uncharacterized protein structures in the PDB that fit the two criteria above. From that dataset of structures, we further uncovered the possible reasons as to why they may have been annotated as proteins with unknown function despite either having been clearly indicated otherwise in the literature or having computationally detectable homologs that are of proteins of known functions.

## 2. Acquisition and Screening of Datasets for Analysis

The dataset of proteins of unknown functions was acquired from the PDB via the advanced search option. The fields used for the advanced search are: (i) Text search containing the phrase “unknown function”; (ii) Experimental method is “X-ray”; and (iii) Macromolecule type is set to “Yes” for protein. Results that possess a header that indicates a molecular function were discarded. A further screen was done to eliminate proteins with annotations in the UniProtKB [5], by first mapping the PDB IDs to UniProtKB IDs, followed by elimination of proteins with available information for the following data fields: Function, Catalytic activity, EC number, and Gene Ontology (GO) based on the restriction that a protein has to have at least 3 GO terms, and other criteria as described below. The remaining proteins formed the dataset used for two levels of homology searching with functionally characterized proteins; first using BLAST [6] for sequence similarity comparison, and then using Dali [7] for structural homology detection.

**Figure 2:**
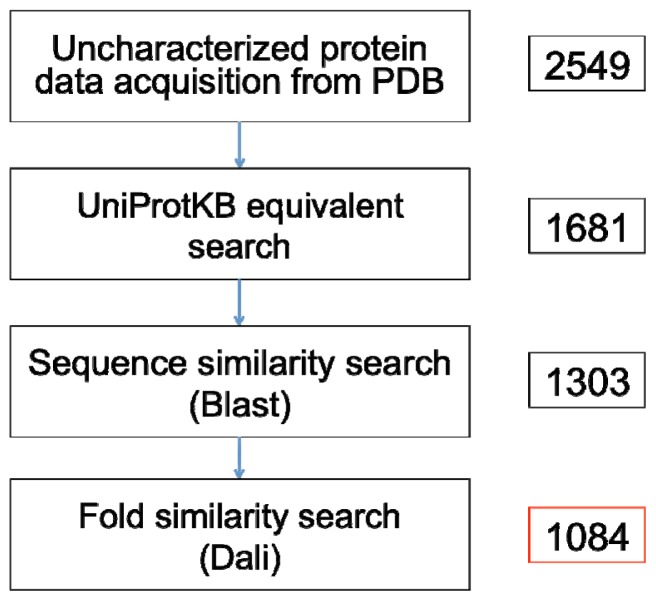
The steps taken to get to a final dataset of 1084 “true” proteins of unknown functions, *i.e.*, PDB entries that lack a significant degree of functional annotation and possess no detectable sequence or structural homology with any protein of known function.

A recent search prior to this paper’s submission on 16 May 2012 using the Advanced Search web interface of the PDB yielded 2549 non-redundant X-ray crystallographic protein structures that are annotated as proteins of unknown function (Figure 2) (Supplementary File). This number included 5 synthetic proteins and were sourced from over 184 different species. Mapping to UniProtKB ID and subsequently checking for functional annotations in the UniProtKB database showed that over 1388 (54.45%) of these proteins already possess various annotations in the UniProtKB. These annotations specifically refer to contents under the “Function” heading, or information of catalytic activity, E.C. number, or Gene Ontology in the UniProtKB. Out of these, we only consider a protein to have sufficient characterization if: (i) it has more than three GO terms; and (ii) the protein name in the UniProtKB does not contain the words “uncharacterized”, “putative”, “unknown”, “predicted”, “unnamed”, “probable”, or “hypothetical”. For the first criteria, if all three terms are from the Cellular Component ontology or all three terms contain the phrase “*-binding”, the characterizations are then considered insufficient. A total of 868 (34.53%) protein structures satisfy the two criteria, whereas 520 structures remained as proteins of unknown functions. Another 1097 proteins have equivalents that are also annotated as uncharacterized proteins in the UniProtKB, and only 64 proteins have no UniProtKB equivalents.

The remaining 1161 proteins with no entry or annotation in the UniProtKB, along with the 520 entries that possess insufficient characterization (Figure 2; Supplementary File), were used as the dataset for sequence similarity searching with BLAST. Deposition dates of the proteins in this dataset range from 13 December 1999 to 20 October 2000. Some of the proteins have been classified into specific families and folds, most being families of proteins with unknown functions and “*-like” family, for example “TT1751-like”. Two species with the most number of uncharacterized proteins in this list are *Thermus thermophilus* and *Bacillus subtilis*.

## 3. Screening at Sequence Similarity Level

A blastp search against the Swiss-Prot portion of the UniProtKB was performed using 1681 queries, with different identity percentage cutoff values for the three domains of Gene Ontology. Our aim was to search for a dataset of proteins that: (i) should be re-annotated; (ii) has a good chance to be re-annotated; or (iii) could be more intensively analyzed computationally or be forwarded for wet laboratory experiments. Based on an assessment of the relationship between the percentage of sequence identity and shared GO terms done in 2007 [8], we only considered an alignment to be significant when the sequence identity is >40% for biological process, >50% for molecular function, and >60% for cellular component, all of which on a large scale study, should indicate a sharing of around 70% of the GO terms for any pair of randomly selected proteins. For the molecular function class, we discarded the hits with the word “*-binding”. We also discarded blast hits that contained names associated with proteins of unknown function as described in the previous step. Other blastp parameters were set to the defaults.

**Figure 3:**
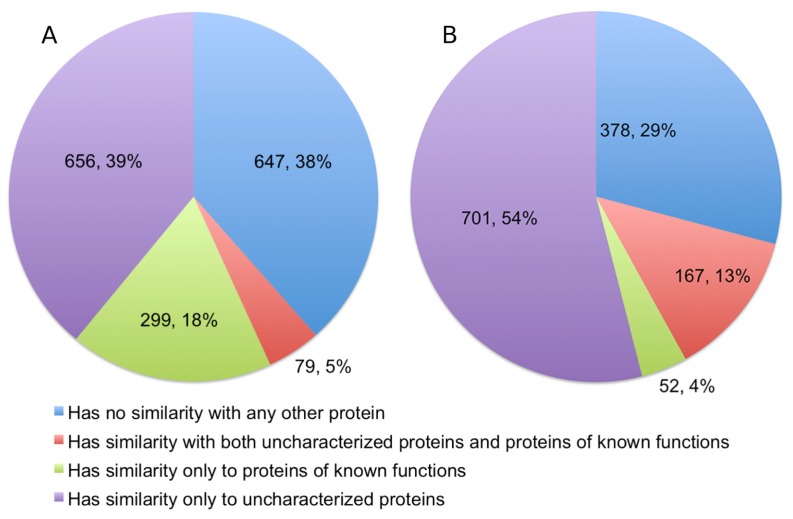
Similarity search results using (**A**) BLAST and (**B**) Dali. Values to the left refer to the number of protein sequences, those on the right refer to the percentage of that number.

The alignments showed that 378 (22.49%) of the queries have significant detectable similarity to functionally characterized proteins in the UniProtKB/Swiss-Prot (Figure 3). Of these, 299 proteins have matches made entirely of characterized proteins, while another 79 proteins have matches that are a mix of uncharacterized and characterized proteins. Quite a number of alignments were discarded based on the restrictions we set for a hit to be considered as characterized. This accounts for 335 (19.93%) sequences in the query. As expected, a significant portion of the queries, numbering at 647 proteins (38.49%), has no similarity to any proteins in the UniProtKB/Swiss-Prot.

An example of a match is the structure with the PDB code 1htw which was deposited on 1 January 2001. Until 2006, this protein structure had no sequence similarity with other proteins of known functions [9]. In 2006 and 2011, two sequences that were annotated as kinases were deposited into the UniProtKB/Swiss-Prot that match the sequence of 1htw at more than 40% identity. Another example is the structure 1jzt that was deposited 17 September 2001. In the years 2007, 2009, and 2012, three different homologous NAD(P)H-hydrate epimerases were integrated into the UniProtKB/SwissProt. However, no annotation pertaining to the possible role of 1jzt as an epimerase is available in the PDB.

## 4. Screening at Structural Folds Similarity Level

When sequence similarity fails to give any insights into the function of an unknown protein, similarity at the fold level is typically sought out and analyzed. For this, we have performed searches on protein structures without any match to functionally characterized protein sequences in UniProtKB/Swiss-Prot using the Dali program. The 1303 proteins remaining after sequence level similarity searching (see Figure 2; Supplementary File), were submitted to the Dali server using the default parameters. Here, we define “functionally characterized proteins” as proteins that possess functional classifications in the PDB that describe the biological or molecular function of a protein. We discarded hits that are classified as proteins of unknown functions or proteins with a header that ambiguously describe a function. We also discarded Dali hits that are in our initial dataset.

The degree of similarity of Dali matches are measured based on the Z-scores of structural alignments. Proteins that are structurally aligned with more than 20 Z-score are “definite homologs”, between 8 and 20 Z-score are “probable homologs”, and less than 8 Z-score are probably unrelated [10]. Therefore, we excluded any results that had a Z-score of less than 20, even when an alignment involves a protein of known function. Structural homology with functionally characterized proteins was detectable for 219 proteins (16.81%), and a further 381 (29.24%) proteins are probable homologs. Therefore, significant fold similarity with functionally characterized proteins was essentially exhibited by 46.05% of the Dali queries, although in the final statistics we only presented the results for alignments with definite homologs (Figure 3).

An interesting example is 1yx1, hypothetical protein PA2260 (deposition date: 19 February 2005), which aligned to various isomerases that were deposited between 2006 and 2008 such as 2hk1, 2qum and 3cqi. Similarly, 2pjz (deposition date: 17 April 2007), which is named as a hypothetical protein in the PDB, has very significant structural similarities with three glucosyl-3-phosphoglycerate synthase, all of which were solved in the year 2012.

## 5. The Missing Annotations for Proteins of Unknown Function in the PDB

One objective of this study was to analyze the occurrences of possible misannotations of uncharacterized proteins in the PDB or legacy classifications and the extent of this situation. We approximate that 23.42% of the proteins in our initial dataset should qualify to be re-annotated or re-assessed based on their significant similarity with functionally characterized proteins. We had chosen to utilize two most commonly used alignment programs at the sequence and structure level—BLAST and Dali—to demonstrate that proteins in our dataset possess detectable sequence or structural similarity to proteins of known functions in the UniProtKB/Swiss-Prot database and in the PDB itself. We propose that this dataset be further studied both *in silico* and in the wet laboratory based on the similarity search results, as these should be relatively easy avenues to assign functions to the proteins in the PDB.

The UniProtKB, which makes use of individually assigned Gene Ontology annotation, should perhaps be the first stop to check for missing annotations in the PDB. Our first step in this survey was to map all the PDB IDs in our dataset to their corresponding UniProtKB IDs, and 868 (34.05%) of their sequence counterparts have various functional annotations in UniProtKB, most of them taken from the “Gene Ontology” section of the individual UniProtKB entry. In cases where the gene ontology is not provided, we checked for any mention of function under the “Function” heading, and we also checked for any mention of catalytic activity or an E.C. number. The UniProtKB is made up of two sections—the manually annotated, reviewed section called UniProtKB/Swiss-Prot, and the unreviewed and automatically annotated UniProtKB/TrEMBL [5]. For a high number of these GO terms, the evidence code shows that the assignment is made on the basis of inference from computational analysis, which can be argued in terms of reliability and might be misannotations. However, in the case of UniProtKB/Swiss-Prot, both experimentally- and computationally-derived functions are curated by human experts, ensuring that the annotations are of high-quality and has been shown to contain close to 0% error [11]. Out of the 868 PDB IDs that were mapped, 404 IDs have sequences that come from the UniProtKB/Swiss-Prot, which means that for almost half of the protein structures that can be mapped to characterized sequences in the UniProtKB, the annotations are dependable and therefore should definitively qualify to put the proteins under specific functional classes in the PDB. As it is, PDB provides a link to GO terms for each entry; however we observed that for these cases, the sequences have been annotated in the UniProtKB but the structures in the PDB are of unknown function. An example is 1l0b, which is thoroughly annotated both in terms of molecular function and biological process in the UniProtKB, but is still classified as a protein of unknown function in the PDB.

Homology-based functional transfer is usually the first technique that is carried out in function prediction attempts due to its simplicity and basic nature. Function is transferred from one sequence or structure to another based on the concept of homology which indicates that two proteins have a common evolutionary origin, and therefore their functions may likely be associated or similar. However, functional transfer based on similarity alone is likely to be insufficient and will possibly contribute to propagation of annotation transfer in the future [11]. Due to the high-throughput nature of the analyses, we abide to the fundamental techniques of functional transfer, with certain cutoff points to minimize possible errors if functional transfers were to be carried out. For the sequence similarity searches using BLAST, our cutoff values were based on the sharing of approximately 70% of the GO terms in a pair of proteins, which is at different sequence identity for the three categories of GO, with the addition of other criteria. For the structure similarity searches, we only considered hits as significant or definite homologs at a very high Z-score of more than 20. For proteins that have not been directly characterized, that is, proteins that possess significant similarity with characterized proteins but with no evidence in the literature, further analyses need to be carried out before their functions can be ascertained. Our aim here was to highlight the existence of such proteins, as the alignments with characterized proteins are very likely to give insights about their functions.

The similarity searches showed that 23% of the Blast queries and 13% of the Dali queries have significant similarity with functionally characterized proteins in the UniProtKB/Swiss-Prot and the PDB, respectively. Our accounting of true uncharacterized proteins in the PDB revealed that the number of proteins that can be rightly claimed as such stands at 1084 entries (Figure 2; see Supplementary File for full list of PDB codes). This number—approximately 43% of the PDB entries annotated as proteins of unknown function—represent PDB coordinates that possess insufficient or no functional characterizations in UniProtKB, and have no detectable sequence or fold similarity to any existing sequence or structures available in the public domain.

As may be expected for a large portion of the probable misannotated uncharacterized proteins, the deposition dates of the hits are later than the deposition dates of the queries and this appears to be the major reason for the lack of functional annotations for this group of proteins. This is true for 43.65% of the Blast alignments, and 69.10% of the Dali alignments. The longest period for a hit to surface on UniProtKB/Swiss-Prot after a structure has been deposited is 10 years, which is for 1fl9, named as “hypothetical protein HI0065” in the PDB (deposition date: 13 August 2000). This structure has a sequence homolog in the UniProtKB—Q66624 (integration date: 8 March 2011), a thymidine kinase. For the Dali alignments, 1kyh (deposition date: 4 February 2002), which is a putative kinase as stated in the PDB in 2002, was deposited 9 years before its Dali hit, 3rqh, a lyase, had its structure deposited (deposition date: 28 April 2011).

Since the hits for half of the Blast alignments and one third of the Dali alignments are deposited into UniProtKB/Swiss-Prot and the PDB before the queries, one would expect these alignments to have been reported in the PDB entries of these queries. The sections or metadata that describe functional annotations in the PDB are class, molecule name, and in many cases the primary citations or literature associated with the structures can be referred to for functional annotation work or assignment. Therefore, these are the sections that we used to check for any mention of the alignments that we have found. We only applied this cross-referencing for Blast and Dali hits that appear in databases before the queries. This is made up of a list of 280 proteins.

The majority of the alignments we have found are, surprisingly, not mentioned in any of the sections mentioned above. We discovered that in some cases, the lack of annotation is attributable to the discrepancies between the sections. For example, some proteins are still classified as proteins of unknown functions although the literature associated to structures have unambiguously described functional annotations. Take for example 1h2h (deposition date: 8 August 2002), which had been described in the literature in 2003 as a dehydrogenase via enzymatic characterization study [12], and 1l6r (deposition date: 13 March 2002), characterized as a new phosphoglycolate phosphatase in 2004 [13]; both proteins are still named as hypothetical proteins and classified as proteins of unknown functions. In other cases, it was found that some proteins are named according to their functions, but are still classified as proteins of unknown functions. For example, 2qen, a “Walker type ATP-ase” with sequence homologs that comprise of other ATP-ases and GTP-ases in UniProtKB/Swiss-Prot, is still classified as a protein of unknown function. There are also cases where even when the literature have reported a function, the names and classes of the proteins are still “hypothetical” or “unknown”. This is true for examples such as 1r8g, classified as a ligase but still holds the name “Hypothetical protein ybdK” and 3eb0, classified as a transferase but is named “Putative uncharacterized protein”.

## 6. Computational Tools to Analyze Proteins of Unknown Functions

Wet laboratory experiments for function determination continues to be a difficult, resource intensive and thus a major limiting step towards function assignment. Bioinformatic analyses can provide insights to assist downstream work in assigning functions to proteins, especially in a high-throughput setting. A variety of software is now available to scrutinize protein sequences and structures for fold and motif similarities. BLAST and Dali are examples of the classical approaches that rely on sequence or fold similarity searching utilized for function inference. Other sequence level tools include PSI-BLAST [14] as well as sequence motif search functions in databases like PROSITE [15], Pfam [16], and InterPro [17]. At the structural level, 3D motifs and patterns can be searched using programs like Profunc [18], SPRITE [19], and PINTS [20]. Other popular methods often deployed to explore and characterize a new structure include algorithms that calculate residue conservation [21] and predict clefts on the protein’s molecular surface [22]. These programs are usually used to supplement results produced by fold and local 3D structure search tools. Phylogenetics and function inference by genomic context, for example by searching for co-expressed genes can also be utilized.

In many cases, similarity at the structure level provides a deeper meaning, due to the 3-dimensional structure being more conserved than sequence [23]. This is shown by a significant number of the proteins in our work, where 77% of our the dataset used for BLAST are proteins that possess no significant sequence similarity with functionally characterized proteins, and out of these, 17% are shown to possess structural homologs of known functions. A case study was carried out using 3hfq, a structure that was found to possess significant similarity with the structure of 1jof, a carboxy-*cis*, *cis*-muconate cyclase, which is a fungal muconate lactonising enzyme with the E.C number 5.5.1.5, at a very high Z-value of 39.5, indicating a clear case of structural homology. At the sequence level, 3hfq shows significant similarity with 6-phosphogluconolactonases and one *cis*, *cis*-muconate lactonizing enzyme I. PSI-BLAST alignment analysis shows that 3hfq, along with 1jof and two other proteins of unknown functions are sequence homologs. A sequence motif search against Interpro showed that it has a significant match to two domains, one domain being a repeated motif that form 7-bladed propellers and the second to the Lactonase family domain (Pfam ID PF10282), whose members comprise of 6-phosphogluconolactonases, carboxy-*cis*, *cis*-muconate cyclases, and muconate cycloisomerases, and members of this family have revealed a 7-bladed β-propeller fold. Structural motif search using SPRITE reveals that the most significant match in terms of RMSD and number of residues is the catalytic motif of 1jof, comprising of residues His 148, Arg 196, Glu 212, and Arg 274, which match respectively to His 144, Arg 190, Glu 206 and Arg of 3hfq. By extracting this motif and searching against the PDB using the program ASSAM [19], it is clear that this motif also superimposes at lower RMSD values with two putative 6-phosphogluconolactonases, 1ri6 and 3scy. Integrating the computational analyses of 3hfq using both sequence and structural tools, it can be inferred that it possesses a 7-bladed beta-propeller fold, and is most probably a 6-phosphogluconolactonase or a carboxy-*cis*, *cis*-muconate cyclase.

It has been clear that functional similarity can exist as an outcome of similar amino acid residues being clustered in 3D space that in turn gives rise to similar chemical activity. Search programs such as ProFunc and SPRITE/ASSAM take this factor into consideration where the target of a search can be directed at finding similar constellations of amino acids thus providing clues to similar chemical activity. At present we see no clear one size fits all program for this purpose because of the different search approaches employed. For example, where some programs superpose the Cα positions in order to find 3D similarities [24,25], others such as SPRITE and ASSAM carry out superpositions of pseudo-atomic representations of the amino acid side chains [19]. This diversity means that for the most part, many of these programs are complementary to each other and it is therefore prudent to at least cover an analysis with conceptually different approaches.

## 7. Conclusions

The number of uncharacterized protein structures that have been experimentally determined and deposited in the PDB remain in the thousands. The structural determination work that was carried out in order to produce these structures has been resource-demanding in terms of funding, time and manpower. Therefore, it is pertinent to ensure that the functional annotations of these proteins are updated regularly following progress in functional characterizations of homologous sequences or structures, or after experimental studies have been undertaken. We propose that a periodic update of function annotations of PDB structures should be carried out, especially when their sequence counterparts are already annotated in sequence databases. Perhaps a semi-automated filtering pipeline can be integrated into the weekly PDB update process where proteins currently classified under “Unknown function” can be re-analyzed, for example by doing alignments with updated data from various databases, for either functional inference or assisting downstream functional experimental strategies or by manual curation. Structures that require further investigations or updates can be flagged for further analytical intervention or for re-annotation by the original depositing authors.
